# Dual EGFR inhibition in combination with anti-VEGF treatment in colorectal cancer

**DOI:** 10.18632/oncoscience.73

**Published:** 2014-08-07

**Authors:** Gerald S. Falchook, Aung Naing, Jennifer J. Wheler, Apostolia M. Tsimberidou, Ralph Zinner, David S. Hong, Siqing Fu, Sarina A. Piha-Paul, Filip Janku, Kenneth R. Hess, Christel Bastida, Razelle Kurzrock

**Affiliations:** ^1^ Drug Development Program, Sarah Cannon Research Institute, Denver, CO 80218; ^2^ Department of Investigational Cancer Therapeutics (Phase I Program), U.T. MD Anderson Cancer Center, Houston, TX; ^3^ Department of Biostatistics, Division of Quantitative Sciences, The University of Texas MD Anderson Cancer Center, Houston, TX; ^4^ Department of Symptom Research, U.T. MD Anderson Cancer Center, Houston, TX; ^5^ Moores Cancer Center, University of California San Diego, La Jolla, CA

**Keywords:** Erolotinib, Cetuximab, Bevacizumab, EGFR, VEGF

## Abstract

Preclinical studies demonstrate that epidermal growth factor receptor (EGFR) signals through both kinase-dependent and independent pathways and that combining a small-molecule EGFR inhibitor, EGFR antibody, and/or anti-angiogenic agent is synergistic. We conducted a dose-escalation, phase I study combining erlotinib, cetuximab, and bevacizumab. The subset of patients with metastatic colorectal cancer was analyzed for safety and antitumor activity. Forty-one patients with heavily pretreated metastatic colorectal cancer received treatment on a range of dose levels. The most common treatment-related grade ≥2 adverse events were rash (68%), hypomagnesemia (37%), and fatigue (15%). Thirty of 34 patients (88%) treated at the full FDA-approved doses of all three drugs tolerated treatment without drug-related dose-limiting effects. Eleven patients (27%) achieved stable disease (SD) ≥6 months and three (7%) achieved a partial response (PR) (total SD>6 months/PR= 14 (34%)). Of the 14 patients with SD≥6 months/PR, eight (57%) had received prior sequential bevacizumab and cetuximab, two (5%) had received bevacizumab and cetuximab concurrently, and four (29%) had received prior bevacizumab but not cetuximab or erlotinib (though three had received prior panitumumab). The combination of bevacizumab, cetuximab, and erlotinib was well tolerated and demonstrated antitumor activity in heavily pretreated patients with metastatic colorectal cancer.

## INTRODUCTION

Epidermal growth factor receptor (EGFR) plays an important role in tumorigenesis [1] and signals via downstream effectors [2]. EGFR protein is overexpressed in 35 to 49% of patients with colorectal cancer [3-5] with a higher percentage of EGFR overexpression in late stage colorectal tumors [6]. Cetuximab, a monoclonal antibody that binds to EGFR [7,8], is approved by the Food and Drug Administration (FDA) for *K-Ras* wild type metastatic colorectal cancer [9]. Erlotinib, a tyrosine kinase inhibitor of EGFR [10], is approved for locally advanced or metastatic non-small cell lung cancer and locally advanced, unresectable, or metastatic pancreatic cancer, but is not currently FDA-approved for colorectal cancer.

Recently, Weihua et al. [11] discovered that EGFR can maintain cancer cell survival independent of its kinase activity. This kinase-independent pathway operates via increased glucose uptake due to stabilization of the SGLT1 glucose transporter, with a downstream effect of reduced autophagy [11]. Furthermore, preclinical studies revealed that combining antibodies and kinase inhibitors was synergistic in lung and head and neck cancer cell lines [12], as well as in lung xenografts [12], and an EGFR-dependent human xenograft model [13]. The combination of cetuximab and erlotinib synergistically inhibits growth of colon cancer cell lines, and has shown antitumor activity in patients with metastatic colorectal cancer [14].

Angiogenesis plays an important role in tumor development and metastasis [15], and is partly mediated by vascular endothelial growth factor (VEGF) [16]. Bevacizumab is a recombinant anti-VEGF monoclonal antibody FDA-approved for treatment of metastatic colorectal cancer in combination with 5-fluorouracil-based chemotherapy [9]. Multiple studies combining chemotherapy and bevacizumab have demonstrated increased efficacy versus chemotherapy alone [17-19]. The addition of bevacizumab to chemotherapy regimens has increased overall survival [17], increased median progression-free survival [18], and improved response rate with longer duration of survival [19] in patients with colorectal cancer.

Anti-VEGF treatment used in conjunction with EGFR inhibitors has shown promise in preclinical and clinical studies. A xenograft study blocking VEGF and EGFR demonstrated synergistic antitumor activity [20], and mice intraperitoneally injected with human colon cancer cells showed improved antitumor activity in response to cetuximab and an anti-VEGF receptor 2 antibody [21]. Phase I and II clinical studies indicate increased efficacy with the combination of anti-VEGF and anti-EGFR therapy, with improved response rate, increased time to progression, and increased overall survival in patients who received cetuximab and bevacizumab [22] versus historical control groups of patients who received cetuximab [23], bevacizumab monotherapy [24], or cetuximab plus chemotherapy [25]. This activity of the combination of cetuximab and bevacizumab may be due to the fact that resistance to EGFR inhibitors is mediated, at least partly, by activating VEGF-dependent signaling [26,27].

Here, we report, for the first time, the results of administering dual EGFR inhibition (erlotinib plus cetuximab) together with an anti-angiogenic agent (bevacizumab) in patients with heavily-pretreated colorectal cancer.

## RESULTS

### Demographics

Forty-one patients with colorectal cancer were enrolled (Table 2). All patients had progressive disease at the time of enrollment. Most patients were heavily pretreated, with a median of five prior therapies (range, 2-12). Thirty-eight patients (93%) had previously received bevacizumab; 33 patients (80%), cetuximab; and one patient (2%) had received no prior study drugs.

**Table 1:** Treatment-related Grade 2-4 adverse events

	Dose Level	1 *n*=2	2 *n*=1	3 *n*=0	4 *n*=1	5 *n*=0	6 *n*=0	7 *n*=3	8 *n*=34	Total *n*=41
	Bevacizumab Dose, mg/kg IV q2w	2.5	5	5	5	7.5	7.5	7.5	10	
	Cetuximab Dose, mg/m^2^ IV weekly*	100, 75	100, 75	200, 125	200, 125	200, 125	400, 250	400, 250	400, 250	
	Erlotinib Dose, mg po daily	50	50	50	100	100	100	150	150	
Skin rash										
Grade 2		0	0	0	1	0	0	2	16	19 (46%)
Grade 3		0	0	0	0	0	0	1	8	9 (22%)
Hypomagnesemia									
Grade 2		0	0	0	0	0	0	0	11	11 (27%)
Grade 3		0	0	0	0	0	0	1	3	4 (10%)
Grade 4		0	0	0	1	0	0	0	2	3 (7%)
Fatigue										
Grade 2		0	0	0	0	0	0	0	5	5 (12%)
Grade 3		0	0	0	0	0	0	0	1	1 (2%)
Diarrhea										
Grade 2		0	0	0	0	0	0	0	3	3 (7%)
Grade 3		0	0	0	0	0	0	1	1	2 (5%)
Hyperbilirubemia										
Grade 2		0	0	0	0	0	0	0	3	3 (7%)
Grade 3		0	0	0	0	0	0	1	0	1 (2%)
Thrombocytopenia										
Grade 2		0	0	0	1	0	0	0	1	2 (5%)
Grade 3^a^		0	0	0	0	0	0	0	1	1 (2%)
Anorexia										
Grade 2		0	0	0	0	0	0	0	2	2 (5%)
Fever and chills										
Grade 2		0	0	0	0	0	0	0	2	2 (5%)
Hypertension										
Grade 2		0	0	0	0	0	0	0	2	2 (5%)
Chest pain										
Grade 3		0	0	0	0	0	0	1	0	1 (2%)
Chills										
Grade 2		0	0	0	0	0	0	0	1	1 (2%)
Constipation										
Grade 2		0	0	0	0	0	0	0	1	1 (2%)
Dyspnea										
Grade 3		0	0	0	0	0	0	1	0	1 (2%)
Fistula										
Grade 3		0	0	0	0	0	0	0	1	1 (2%)
Hand and foot syndrome										
Grade 2		0	0	0	1	0	0	0	0	1 (2%)
Increased AST										
Grade 2		0	0	0	0	0	0	0	1	1 (2%)
Increased AST/ALT										
Grade 2		0	0	0	0	0	0	1	0	1 (2%)
Infusion reaction										
Grade 3		0	0	0	0	0	0	1	0	1 (2%)
Neutropenia										
Grade 3		0	0	0	0	0	0	0	1	1 (2%)
Proteinuria										
Grade 2		0	0	0	0	0	0	0	1	1 (2%)
Pruritis										
Grade 3		0	0	0	0	0	0	0	1	1 (2%)

Abbreviations: DLT, dose-limiting toxicity; IV, intravenous; po, orally

† Recommended Phase II dose.^31^ This includes full approved doses of each drug.

* Cetuximab dose shown as loading dose, maintenance dose.

**Table 2:** Patient Demographics

Characteristics (*n*=41)	
Age (years)	
Median	57
Range	32-76
Gender, *n* (%)	
Men	19 (46%)
Women	22 (54%)
Histologies, *n* (%)	
Adenocarcinoma	41 (100%)
No. of prior systemic therapies	
Median	5
Range	2-12
Prior systemic treatment	
Prior bevacizumab	38 (93%)
Prior bevacizumab (but no prior cetuximab or erlotinib)	7 (17%)
Prior bevacizumab and cetuximab (sequential)	27 (66%)
Prior bevacizumab and cetuximab (concurrent)	4 (10%)
Prior cetuximab	33 (80%)
Prior cetuximab (but no prior bevacizumab or erlotinib)	2 (5%)
Prior erlotinib	0 (0%)
Prior panitumumab	5 (12%)
*KRAS* mutations, *n* (%)	
Positive	2 (5%)
Negative	31 (76%)
Unknown	8 (20%)
*EGFR* mutations, *n* (%)	
Positive	1 (2%)
Negative	16 (39%)
Unknown	24 (59%)
*P53* mutations, *n* (%)	
Positive	6 (15%)
Negative	0 (0%)
Unknown	35 (85%)
*BRAF* mutations, *n* (%)	
Positive	1 (2%)
Negative	27 (66%)
Unknown	13 (32%)
*PIK3CA* mutations, *n* (%)	
Positive	3 (7%)
Negative	18 (44%)
Unknown	20 (49%)
ECOG performance status, *n* (%)	
0	5 (12%)
1	34 (83%)
2	2 (5%)

Abbreviation: ECOG, Easter Cooperative Oncology Group; EGFR, epidermal growth factor receptor 1; P53, turmor protein 53.

### Adverse Events

The most common treatment-related grade 2 or higher adverse events were rash (n=28, 68%), hypomagnesemia (n=18, 44%), fatigue (n=6, 14%), diarrhea (n=5, 12%), and hyperbiliruemia (n=4, 10%) (Table 1). Eight patients (20%) experienced no drug-related toxicity higher than grade 1. Seventeen patients (41%) required a dose reduction because of toxicity, including cetuximab in 13 patients for rash, two patients for diarrhea, one patient for hypomagnesemia, and one patient for diarrhea and transaminitis. Two patients (5%) withdrew due to toxicity, including grade 2 skin rash in cycle 1 (n=1) and grade 2 diarrhea and fatigue in cycle 1 (n=1). No deaths resulted from adverse events. The RP2D was level 8, which include the recommended FDA-approved full doses of each medication [28].

### Responses and time to treatment failure

**Figure 1:**
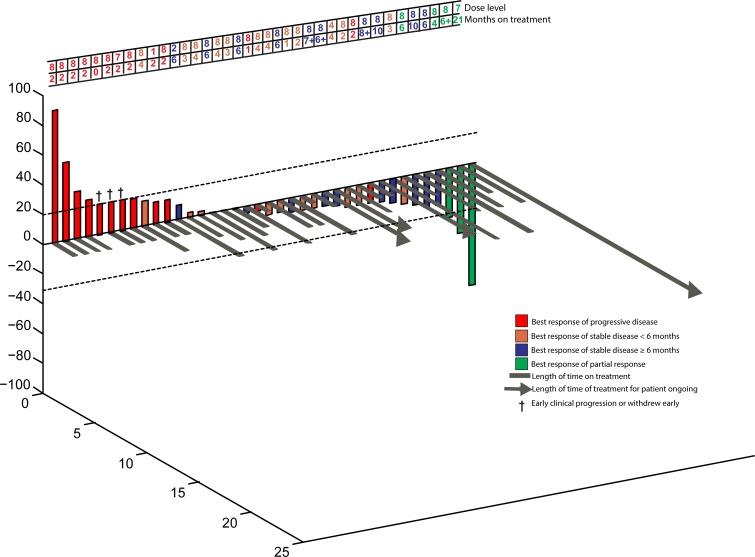
3D-Waterfall Best response in 38 colorectal cancer patients treated. Patients with early clinical progression or new lesions before first restaging are indicated arbitrarily as +21% and are marked with a “†”. Three patients who withdrew early before restaging because of toxicity (n=2) or financial reasons (n=1) are not depicted in the figure. Patients with progressive disease are shown in red; patients who achieved stable disease are shown in orange, patients who achieved stable disease of at least six months are shown in blue; patients who achieved partial response are shown in green. The dose level and treatment duration (months) for each patient are shown in the table below. Patients still on treatment have a “+” after the number of months and are indicated with an arrow (>) on the grey bar for that patient.

**Figure 2:**
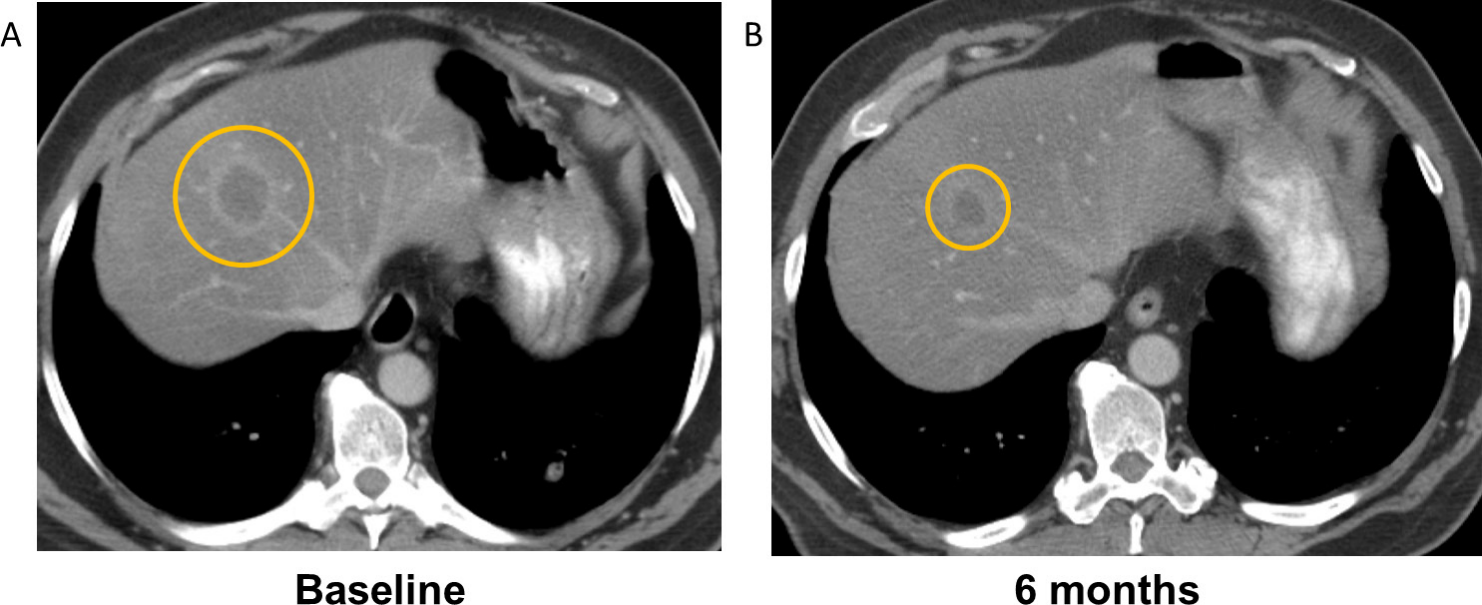
Computerized tomography (CT) demonstrating response to treatment with combination cetuximab, erlotinib, and bevacizumab in a patient with *KRAS* wild-type colorectal cancer who had received prior cetuximab and bevacizumab A decrease in tumor size of 45% by RECIST was observed, and the patient received treatment for 6 months. Panel A demonstrates a liver metastasis at baseline, and Panel B demonstrates the tumor after 6 months of treatment.

In total, SD≥6 months or PR was achieved in 14 patients (34%) (Figure 1). The overall confirmed response rate was 7% (PR). Eleven patients (27%) achieved stable disease (SD) lasting at least 6 months (duration was 6, 6, 6, 6, 6, 6, 6+, 7+, 8+, 10 and 10 months). Three patients (7%) achieved a PR and received treatment for 4, 6+, and 21 months (Table 3, Figure 2). Two patients withdrew before the first restaging assessment due to toxicity, and one patient withdrew early because of financial considerations. However, all patients were considered eligible for evaluation of response. Exploratory analysis of genomic aberrations was performed in selected patients who had tissue available (Table 4).

**Table 3:** Patient characteristics for those who achieved stable disease of at least 6 months, partial response, or complete response

Case #	Best Response %	Treatment duration (months)	KRAS mutation	PTEN	TP53 mutation	EGFR mutation	HER2 Amplification	PIK3CA mutation	Prior bevacizumab	Prior cetuximab	Prior panitumumab	Brain metastases	Dose Level	Rash Grade
135	−81	21	NO	ND	ND	ND	ND	ND	YES	NO	NO	NO	7	2
336	−44	6+	NO	ND	YES	NO	NO	NO	YES	NO	NO	NO	8	3
291	−33	4	NO	ND	ND	NO	ND	NO	YES	YES	NO	NO	8	2

171	−23	6	NO	ND	YES	NO	ND	NO	YES	YES	NO	NO	8	1
215	−20	6	NO	ND	ND	ND	ND	ND	YES^a^	YES^a^	NO	NO	8	2
245	−16	10	NO	PRESENT	ND	NO	ND	NO	YES	YES	NO	NO	8	3
314	−14	8+	NO	ND	ND	ND	ND	ND	YES	YES	NO	NO	8	2
335	−11	6+	NO	ND	ND	ND	ND	ND	YES	NO	YES	NO	8	1
235	−11	10	NO	LOSS	YES	ND	ND	NO	YES	YES	NO	NO	8	3
327	−9	7+	NO	ND	ND	NO	ND	ND	YES	NO	YES	NO	8	1
277	−9	6	NO	ND	ND	ND	ND	ND	YES	YES	NO	NO	8	2
260	−4	6	NO	PRESENT	ND	ND	ND	YES	YES	YES	NO	NO	8	2
221	0	6	ND	ND	ND	ND	ND	ND	YES	YES	NO	NO	8	3
22	10	6	ND	ND	ND	ND	ND	ND	YES	YES	NO	NO	2	0

Abbreviations: EGFR, epidermal growth factor receptor 1; HER2, human epidermal growth factor receptor 2; ND, not done; PIK3CA, phosphoinositide-3-kinase, catalytic, alpha polypeptide.

a indicates patients who received prior study drugs concurrently.

+ indicates ongoing therapy

**Table 4:** Gene mutation status and response

Gene	Proportion of patients with mutation out of number tested (% of patients positive)	Mutations identified	Number of patients who achieved SD>6 months/PR (by mutation status)	
			Mutant	Wild type
*EGFR*	1 of 17 (6%)	G719D (*n*=1)	EGFR mutant: 0/1	EGFR wt: 5/16
*BRAF*	1 of 28 (4%)	V600E (*n*=1)	BRAF mutant: 0/1	BRAF wt: 8/27
*KRAS*	2 of 33 (6%)	G12V (*n*=1)G12D (*n*=1)	KRAS mutant 0/2	KRAS wt: 13/31
*P53*	6 of 6 (100%)	R175H (*n*=3)T125K (*n*=1)S227fs*1 (*n*=1)R248W (*n*=1)	P53 mutant: 3/6	N/A
*PIK3CA*	3 of 21 (14%)	R1023Q (*n*=1)E545K (*n*=1)Q546R (*n*=1)	PIK3CA mutant: 1/3	PIK3C wt: 6 of 18
*PTEN*	1 of 6 (17%) had PTEN loss 2 of 6 (33%) had weakly present PTEN 3 of 6 (50%) had PTEN present	N/A	1 of 1 (PTEN loss)	2 of 3 (PTEN present)
*FBXW7*	1 of 1(100%)	R222 (*n*=1)	0 of 1	N/A
*APC*	1 of 1(100%)	T820fs*7 and P1439fd*34 (*n*=1)	0 of 1	N/A

**Figure 3:**
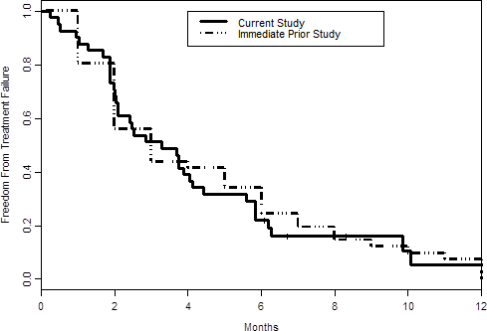
Kaplan-Meier curve for time to treatment failure for the current study versus the immediately prior standard therapy

Median time to treatment failure for the current treatment is 3.3 months with 95% CI = (2.1, 4.4), while for the immediately prior standard treatment, the median is 3.0 (2.0, 6.0). (p=.71) (Figure 3).

### Prior EGFR inhibitor or VEGF Inhibitor Therapy and Response

Of 41 patients on study, a total of 38 patients (93%) had received prior bevacizumab, and a total of 33 patients (80%) had received prior cetuximab (Table 2). Thirty-one patients (76%) had received prior bevacizumab and prior cetuximab (27 sequentially, 4 concurrently), seven patients (17%) had received prior bevacizumab and no other study drugs, and two patients (5%) had received prior cetuximab and no additional study drugs. No patients had received prior erlotinib. Four patients had previously received panitumumab, a monoclonal antibody that binds to EGFR and inhibits epidermal growth factor autocrine signaling.

Prior bevacizumab and cetuximab, even if given concurrently, did not preclude SD≥6 months/PR. Four of the seven patients (56%) who received prior bevacizumab and no other prior study drugs achieved SD≥6 months/ PR; three of these four patients had also received prior panitumumab. Of the 38 patients who had received prior bevacizumab, 14 (37%) achieved SD≥6 months/PR; of the 33 patients that received prior cetuximab, 10 (30%) achieved SD≥6 months/PR; of the 31 patients who had received prior cetuximab and bevacizumab, 10 (32%) achieved SD≥6 months/PR. The latter included four patients who had received prior concurrent bevacizumab and cetuximab, two of whom achieved SD≥6 months/PR. Of the 14 patients with SD≥6 months/PR, eight (57%) had received prior sequential bevacizumab and cetuximab, two (5%) had received bevacizumab and cetuximab concurrently, and four (29%) had received prior bevacizumab but not cetuximab (though three had received prior panitumumab (Table 3 and Figure 1). Patient 135, who had received prior panitumumab and had also previously received bevacizumab, achieved a partial response and was on study for 21 months (Table 3).

### Dosing and Response

Of 37 patients on dose levels 7 or 8, thirteen (35%) achieved SD≥6 months/PR. Of the four patients treated at dose levels 1-6, one patient (25%) achieved SD≥6 months/PR (Table 1 and Figure 1). There was no obvious dose-response correlation, although the number of patients at lower dose levels was small.

### Toxicity and Response

Rash was the most frequently observed toxicity in patients (Table 1). Patients with grade 2 or higher rash were not significantly more likely to attain SD≥6 months/PR (two-tailed chi squared, p=0.76). Nineteen patients experienced grade 2 rash, of whom six (31%) achieved SD≥6 months/PR. Four of nine patients (44%) with grade 3 rash achieved SD≥6 months/PR (Table 3). Of 13 patients with grade 1 or no rash, four (31%) achieved SD≥6 months/PR.

## DISCUSSION

We report the results of the cohort of patients with colorectal cancer treated on a phase I dose-escalation trial of combination cetuximab, erlotinib, and bevacizumab. The rationale for this combination was: (1) preclinical and clinical studies that suggested increased activity when anti-VEGF therapy was combined with EGFR inhibitors [20,21], (2) preclinical studies indicating that EGFR signals through both kinase-dependent and -independent pathways [11], and (3) clinical trials demonstrating increased overall survival in patients treated with cetuximab and bevacizumab [22].

This combination of drugs was well-tolerated. The RP2D was determined to be the full FDA-approved doses for all three drugs [28], and 31 of the 34 patients (91%) treated at the RP2D tolerated treatment without drug-related dose-limiting effects.

This regimen demonstrated antitumor activity in patients with colorectal cancer, including 14 patients (34%) who had a best overall response of SD≥6 months (n=11) or PR (n=3). SD≥6 months/PR was observed even in patients who had received prior bevacizumab and/or cetuximab or treated at a lower dose level.

Previous phase II/III clinical studies combining cetuximab and bevacizumab with cytotoxic chemotherapy in patients with colorectal cancer resulted in disappointing results [29-32]. One notable difference is that our current study included erlotinib dosing up to 150 mg daily, in contrast to 100 mg in prior studies [14]. It is conceivable that, in this context, the chemotherapy component of the above regimen may be detrimental, whereas regimens that combine anti-EGFR and anti-VEGF agents without cytotoxic chemotherapy deserve further investigation. Indeed, studies in pancreatic adenocarcinoma [33] and squamous cell carcinoma of the head and neck [34] that use a combination of cetuximab and bevacizumab show promising results.

A prior preclinical study combining erlotinib and cetuximab demonstrated synergistic antitumor activity in colorectal cancer [14], and a related phase II clinical trial of this combination achieved an overall response rate of 31% [14], which is similar to the rate of SD≥6 months/PR observed in our study. Importantly, the phase II study of erlotinib and cetuximab did not report the number of prior systemic therapies, whereas our study included patients who were heavily pretreated (median of five prior systemic therapies).

Remarkably, patients in our study who previously had failed bevacizumab and/or cetuximab were able to acheive SD≥6 months/PR. Recent studies suggest that combining EGFR kinase inhibitors and anti-EGFR antibodies may be more effective than either alone, perhaps because EGFR is able to maintain cancer cell survival independent of its kinase activity [11-14]. The clinical data presented here also support combining kinase inhibitors and antibodies.

Exploratory analysis of molecular aberrations was performed. Of the 14 patients who achieved SD≥6 months/PR in our study, four had mutations present (PIK3CA E545K (n=1), TP53 R175H and PTEN loss (n=1), TP53 R175H (n=1), TP53 R248W (n=1). This analysis is limited however by the fact that only a small number of mutations were evaluated in individual patients.

Previous studies demonstrated a correlation between rash and response to EGFR inhibitors [35,36]. However, in our study we did not observe a trend of higher grade rash in patients with SD≥6 months/PR (p=0.76). Larger studies could have more definitive conclusions in this regard.

There are several limitations to this study. First, molecular correlates could only be obtained in a small subset of patients, precluding a robust analysis. Second, these patients had a median of five prior therapies in the metastatic setting, perhaps attenuating their ability to respond. Third, determination of time to treatment failure of prior therapy was obtained from chart review, rather than prospectively.

In conclusion, the results presented here demonstrate that dual inhibition of EGFR with erlotinib and cetuximab, combined with the VEGF antibody bevacizumab, is well-tolerated, allowing full doses of all three drugs in patients with colorectal cancer. The most common side effect is rash. SD≥6 months/PR was achieved in 34% of this heavily pretreated patient population, including patients treated with prior bevacizumab and/or cetuximab. These findings merit further investigation in a larger study of patients with metastatic colorectal cancer.

## METHODS

### Study Design

This report is a subset analysis of a larger phase I study of combination cetuximab, erlotinib, and bevacizumab. The study was conducted at The University of Texas M. D. Anderson Cancer Center (MDACC) per Institutional Review Board guidelines. The colorectal cancer cohort reported herein included all patients with colorectal cancer who started therapy between 12/10/2007 and 5/7/2012 as part of a dose-escalation study conducted in patients with advanced cancer. The dose escalation portion of the study determined the recommended phase II dose (RP2D) to be bevacizumab 10 mg/kg IV every two weeks; cetuximab loading 400 mg/m^2^, maintenance 250 mg/m^2^ IV weekly; and erlotinib 150 mg PO daily on a 28 cycle [28]. Patients were treated at variable dose levels, depending on the time of study entry (Table 1).

### Patients

Patients had metastatic or advanced colorectal cancer not amendable to standard therapy, an Eastern Cooperative Oncology Group (ECOG) performance status 0-2 [37], and adequate hematologic, hepatic, and renal function. Exclusion criteria included hemoptysis, unexplained bleeding, significant cardiovascular disease, intercurrent uncontrolled illness, significant gastrointestinal bleeding within 28 days, hemorrhagic brain metastases, prior abdominal surgery within 30 days, pregnancy, and a history of hypersensitivity to bevacizumab, cetuximab, and/or erlotinib. Treatment with prior cytotoxic therapies must have ended at least three weeks prior to enrollment, and biologic therapy must have either ended at least two weeks or five drug half-lives prior to enrollment, whichever was shorter. Patients may have received an unlimited number of prior therapies, including prior anti-EGFR and anti-angiogenic agents.

### Safety

Clinically significant adverse events were assessed according to the National Cancer Institute Common Terminology Criteria for Adverse Events (NCI CTCAE), version 3.0. History, physical exam, hematology, blood chemistry, and urinalysis were performed at baseline and regular intervals while receiving treatment.

### Evaluation of Efficacy

Treatment efficacy was evaluated by diagnostic imaging per Response Evaluation Criteria in Solid Tumors (RECIST) 1.0 [38]. Radiologic assessments were conducted at baseline and about every 8 weeks thereafter.

### Molecular Testing

*EGFR, KRAS, PIK3CA, p53*, and *BRAF* mutation analysis, as well as PTEN expression by immunohistochemistry, were performed in the Clinical Laboratory Improvement Amendments (CLIA)-approved MDACC laboratory for patients with available archived tissue (supplementary methods).

### Statistical Analysis

Analyses were descriptive and exploratory. Correlational statistics were determined by Spearman's correlation and dichotomous variables were evaluated with chi-square or Fisher's exact test. We estimated the time to treatment failure distribution using the Kaplan-Meier product limit method, and estimation of 95% confidence interval for the mean was calculated using conventional methods (mean +/− 2*SEM where SEM = standard error of the mean). Time to treatment failure was defined as the duration of treatment received until a patient developed progressive disease or withdrawal from study because of toxicity or any other reason.

## SUPPLEMENTARY METHODS


