# Supplementary material for: Dual EGFR inhibition in combination with anti-VEGF treatment in colorectal cancer
Source: Oncoscience. 2014 Aug 7;1(8):540–9. doi: 10.18632/oncoscience.73 (PMC4278330; doi:10.18632/oncoscience.73)
Supplement: Supplementary file 1 [file oncoscience-01-0540-s001.docx]

**Supplementary methods**

For *EGFR* (exons 18-21 of the kinase domain), *KRAS* (codons 12, 13 and 61), *PIK3CA* (codons 532-554 in exon 9 and codons 1011-1062 in exon 20), *p53* (exons 4-9), and *BRAF* (codons 595 to 600 of exon 15) testing, PCR-based sequencing analysis was performed on DNA extracted from paraffin-embedded tumor tissue. The lower limit of detection was approximately one cell bearing the mutation per five to ten normal cells. PTEN expression was determined by immunohistochemistry using anti-PTEN monoclonal mouse antibody (Dako, Carpinteria, CA).
